# Anti-corruption, transparency and accountability in health: concepts, frameworks, and approaches

**DOI:** 10.1080/16549716.2019.1694744

**Published:** 2020-03-20

**Authors:** Taryn Vian

**Affiliations:** School of Nursing and Health Professions, University of San Francisco, San Francisco, CA, USA

**Keywords:** Anti-Corruption, Transparency and Accountability, Corruption, fraud, ethics, compliance, governance, health systems strengthening

## Abstract

**Background**: As called for by the Sustainable Development Goals, governments, development partners and civil society are working on anti-corruption, transparency and accountability approaches to control corruption and advance Universal Health Coverage.

**Objectives**: The objective of this review is to summarize concepts, frameworks, and approaches used to identify corruption risks and consequences of corruption on health systems and outcomes. We also inventory interventions to fight corruption and increase transparency and accountability.

**Methods**: We performed a critical review based on a systematic search of literature in PubMed and Web of Science and reviewed background papers and presentations from two international technical meetings on the topic of anti-corruption and health. We identified concepts, frameworks and approaches and summarized updated evidence of types and causes corruption in the health sector.

**Results**: Corruption, or the abuse of power for private gain, in health systems includes bribes and kickbacks, embezzlement, fraud, political influence/nepotism and informal payments, among other behaviors. Drivers of corruption include individual and systems level factors such as financial pressures, poorly managed conflicts of interest, and weak regulatory and enforcement systems. We identify six typologies and frameworks that model relationships influencing the scope and seriousness of corruption, and show how anti-corruption strategies such as transparency, accountability, and civic participation can affect corruption risk. Little research exists on the effectiveness of anti-corruption measures; however, interventions such as community monitoring and insurance fraud control programs show promise.

**Conclusions**: Corruption undermines the capacity of health systems to contribute to better health, economic growth and development. Interventions and resources on prevention and control of corruption are essential components of health system strengthening for Universal Health Coverage.

## Background

The UN Office of the High Commissioner on Human Rights recognizes corruption as an ‘enormous obstacle to the realization of all human rights,’ and advocates transparency, accountability, non-discrimination and meaningful participation as effective means to fight corruption [1,2]. Corruption fundamentally undermines good governance, weakens health systems, and violates human rights. It also disrupts progress toward the goal of Universal Health Coverage (UHC), the principle that all individuals and communities should be able to access the essential health services they need, without financial hardship [3]. Governments, development partners, and health researchers are promoting anti-corruption efforts to control corruption in health systems across the globe; however, existing research does not provide a comprehensive picture of how these efforts align. It is therefore critical to look across the different frameworks used and consider applicability of strategies in different settings. This can help to advance a coherent agenda to prevent corruption through health systems strengthening. Table 1 defines the key concepts of anti-corruption, transparency, and accountability (ACTA). These definitions, and the relationships among these concepts, are discussed below.

**Table 1.** Definitions.

Term	Definition
Corruption	Abuse of entrusted power for private gain. This includes bribes, embezzlement, misappropriation, diversion of government property, trading in influence, abuse of function, and illicit enrichment.
Fraud and abuse	Fraud: Illegally obtaining a benefit of any nature by intentionally breaking a rule. Abuse: unjustly obtaining a benefit of any nature by knowingly stretching a rule or by taking advantage of an absence of rule. Includes reimbursement fraud, procurement fraud, unauthorized absenteeism, ghost workers, and other forms.
Transparency	Transparency is a public value that requires that citizens be informed about how and why decisions are made, including procedures, criteria applied by government decision makers, the evidence used to reach decisions, and results. Often transparency refers to access to information.
Accountability	Accountability is a public value that requires government institutions to explain and make understandable their performance in achieving goals and addressing the needs of the public, in comparison to standards and commitments. It requires visible, responsive action if standards and commitments are not met.

Sources: Transparency International www.transparency.org; UN Convention Against Corruption https://www.unodc.org/unodc/en/treaties/CAC/; Sommersguter-Reichmann, et al., 2018; Vian, et al., 2017; Paschke, et al., 2018.

### Anti-corruption

Corruption is defined as abuse of entrusted power for private gain [4]. Healthcare fraud and abuse are often included in the discussion of corruption, as these practices often involve abuse of power [5]. Anti-corruption, therefore, comprises actions taken to prevent, curb, or oppose corruption, and to mitigate its negative impacts.

Practices defined as corruption may vary depending on country and context. Hence, anti-corruption efforts can take multiple forms depending on the situation. For example, one study in Brazil defined corruption to include diversion of federally transferred resources from municipal bank accounts, claim of purchases that never actually occurred, over-invoicing goods and services at a value above marketplace, and irregular public procurements marked by illegal call-for-bids in any respect (such as the concession of the contract to a family member’s firm) [6]. The UN Convention Against Corruption (UNCAC), specifies that signatory countries must criminalize specific forms of corruption, including bribery of national and foreign public officials, embezzlement, misappropriation, diversion of property by public officials, trading in influence, abuse of functions, and illicit enrichment [7]. While this treaty sets internationally agreed standards, country compliance varies [8].

### Transparency

Transparency refers to the public availability of usable information. This may mitigate corruption risks as it allows scrutiny of public actors and their decisions. Governments have an obligation to provide clarity on the rules and results of health care delivery processes and to reveal any secondary interests that may influence decisions of health care providers and policy makers [9,10]. Transparency is considered a necessary, though not sufficient condition for accountability and the prevention of corruption [11].

### Accountability

Accountability refers to the obligation of those in power to explain, make understandable, and take responsibility for their decisions, actions, and performance. Officials are responsible for acting according to standards and commitments made public in the form of laws, regulations, guidelines, procedures, and policies [10]. Experts note three elements of accountability: 1) answerability, or the obligation to justify one’s action; 2) enforcement, the consequences imposed if the action and justification are not satisfactory; and 3) responsiveness, the willingness of those held accountable to respond to demands made [12]. Enforcement must impose consequences on the corrupt agent that are large enough to deter corruption [13]. Social accountability can include grievance procedures and social audits to collect qualitative and quantitative data on citizen opinions about and lived experiences with services [14].

### Linkages among concepts

Corruption thrives in settings where agents are able to bend or break rules without being detected, either because the rules themselves are not codified in writing, are ambiguous, or are not made public, or because performance measurement, monitoring, and enforcement are lacking. Lambert-Mogiliansky (2015) highlights the links between transparency, accountability, and corruption, stating that ‘In the absence of any signal of the official’s behavior (e.g. a performance measure, a verification outcome, announcements, and service users’ complaints) citizens have no way of preventing a corrupt official from diverting money: the official is in effect not at all accountable for the use of resources’ [12]. When policies, entitlements, procedures, and performance measures are transparent, observers can detect improper behavior more easily. The greater the transparency, the more space for government officials to be held accountable for their actions [15]. Transparency applied to processes can make citizens aware of government commitments (expressed through policies and plans), and the targets against which government performance should be measured. Transparency applied to service delivery can help citizens and government oversight agencies to know where performance is falling short, and to ask government agents to answer for the performance deficits.

Although transparency is a necessary input, it is also important to consider how to share information, and with whom, to best facilitate accountability. For example, a country might undertake an annual budget survey to measure the proportion of people who had a health concern but did not seek care for financial reasons. Data analyzed over time might show that the state is making progress on this measure (i.e. increasing financial access), but that the progress was heavily weighted toward urban populations. Having this regularly collected, publicly available measure of financial access to care can allow the government and citizens to monitor progress toward UHC goals, and highlight remedial actions which could improve performance such as targeted efforts to reduce out-of-pocket payments (including informal payments or bribes) among rural residents.

## Objectives

The objective of this critical review is to summarize ACTA concepts, frameworks, and approaches used to measure health corruption, and to examine the consequences of corruption on health systems and outcomes. The review also examines ACTA interventions and strategies to reduce corruption risks. It is timely to provide a current picture of how the situation has changed in recent years using updated information. Thus, we update an earlier review published in 2008 by Vian, which also looked at frameworks and methodologies to measure corruption risks in health systems [16]. Using recent literature, we examine evidence on interventions and propose future directions for research.

## Methods

Our study design was a critical review to analyze material from diverse sources including a systematic search of peer-reviewed literature and documentation from two international technical meetings [17]. For the peer-reviewed literature, we searched for articles within PubMed and Web of Science using the keywords: corruption AND health AND (framework OR model OR theory). Our search was limited the years 2008 to 2018, and to studies written in English. After removing duplicates, the search generated 82 abstracts. We retained references that described corruption problems or consequences or provided evidence of the association of corruption and health outcomes, while removing editorials, commentaries, and letters. Following the initial screening, 73 abstracts remained. We then reviewed the full texts and excluded articles with only casual referencing of corruption in the health sector or that did not propose theoretical constructs or provide empirical evidence. This resulted in 46 articles. Appendix A provides a summary of these citations.

This paper also draws on insights from two expert technical meetings and related background papers and presentations commissioned by the World Health Organization (WHO) on the topic of anti-corruption, transparency and accountability in the health sector. The meetings held November 2017 and March 2018 in Geneva provided a unique opportunity for technical discussions among health and corruption experts working in this field. Appendix B includes the list of attendees and references.

Our analysis was guided by the themes included in the original review of corruption in the health sector mentioned above (Vian, 2008), including measures of corruption in the health sector, evidence of consequences, theoretical frameworks, and anti-corruption approaches [16].

In the next section, we present our findings on measuring corruption (including updated evidence for key types of corruption); consequences (how corruption and lack of transparency and accountability undermine health); frameworks and models for corruption, transparency and accountability; and anti-corruption approaches (interventions and evidence of effectiveness). We conclude with areas for further research.

## Results

### Measuring corruption

Corruption is heterogeneous and multiple measures can help triangulate the problem [18]. Corruption in health is measured through surveys of attitudes and experience [19–22], audits and special studies [23–25], and complaints or investigative reporting [26,27]. Proxy measures such as prices may indicate procurement corruption [28]. Qualitative studies of corruption perceptions help inform prevention efforts [29–31].

**Figure 1.**
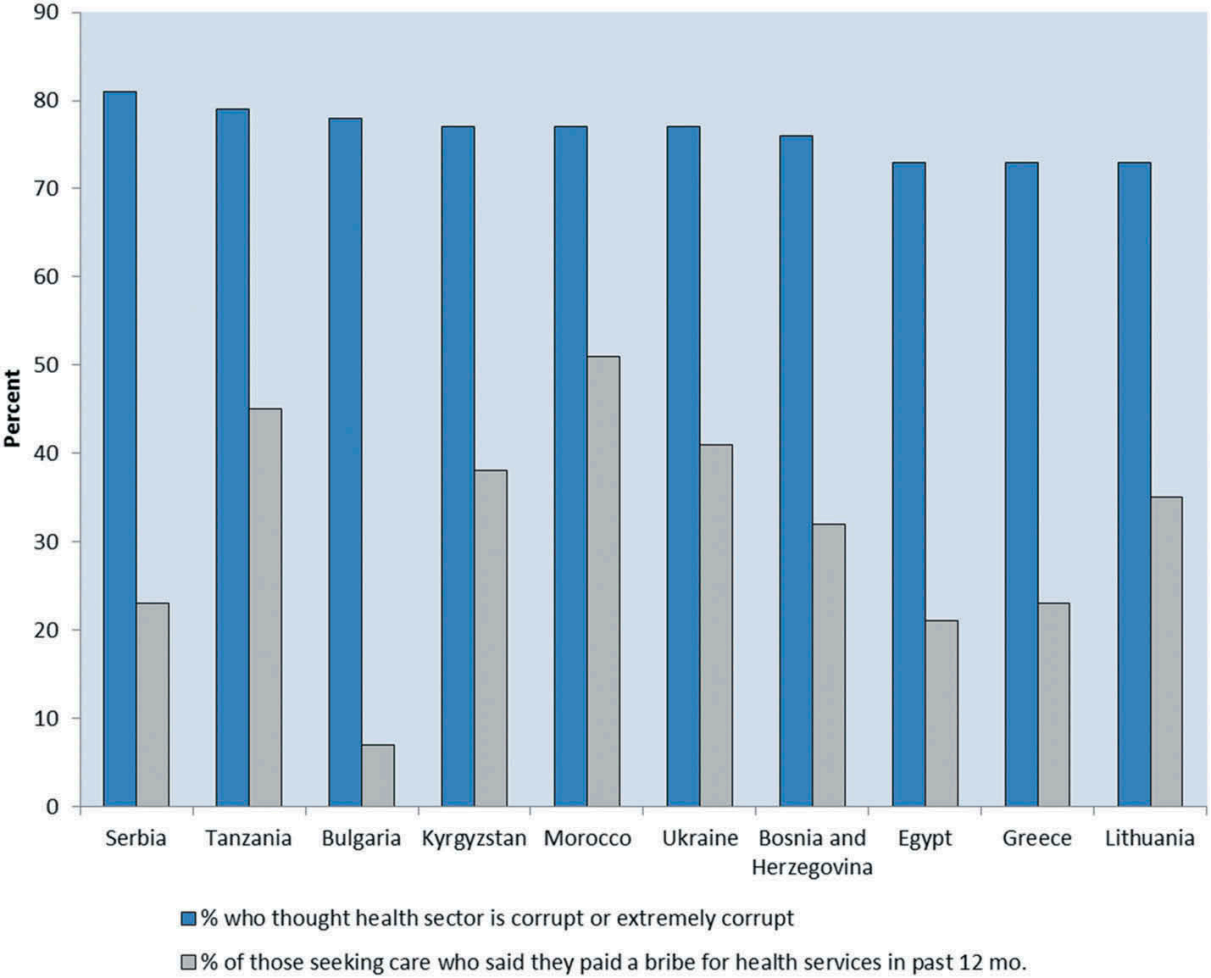
Countries with highest percentages of respondents reporting perceptions that the health sector is corrupt or extremely corrupt and reporting payment of a bribe in the past 12 months. Source: Transparency International, 2013. Study included 107 countries. Albania and Russia, which had high perceived health sector corruption, did not have bribe data, and therefore are not shown.

In Transparency International’s Global Corruption Barometer (GCB) survey [32], two questions relate to health: 1) respondents are asked about perceptions of corruption, and 2) for those who sought health care services in the past year, respondents are asked whether they paid a bribe (Figure 1).

Such surveys can show changes over time. For example, in Nicaragua, public perception of corruption and the proportion of people who reported having made an informal payment for health services declined over time, although the decline in perceptions lagged behind experience [33]. Respondents’ perception that community opinions mattered to municipal government officials was strongly associated with not believing the government was corrupt. Transparency appeared to engage community trust: being informed about government use of funds was associated with more positive community opinions [33].

Specific types of corruption have been measured in more detail, including informal payments, ghost workers, absenteeism, dual practice, health insurance fraud, and substandard and falsified medicines. We summarize evidence on the scope and nature of these problems below.

#### Informal payments

Informal payments occur in many regions and rates vary widely [22,34,35]. A recent systematic review of 38 studies on methodology and burden of informal patient payments found that 2–80% of respondents had made informal payments [22]. Regional data on informal payments are tracked through public attitude survey polls conducted by non-partisan research institutions over time, such as the Afrobarometer and the European Commission’s Eurobarometer program. Analysis of Afrobarometer polls from 2011–2013 in 33 countries in Africa found that the proportion of respondents who paid a bribe in the last 12 months at a public health facility was over 40% in Liberia, Sierra Leone, Guinea, Egypt and Morocco, and less than 5% in Botswana, Cape Verde, Lesotho, Malawi, Mauritius, Namibia and Swaziland [36]. According to the Eurobarometer 470 report conducted in 2017, European countries with the highest informal payment rates included Romania (19%), Hungary (17%), Greece (13%), and Lithuania (12%) [37].

Few studies have tried to separate the burdens posed by different types of payments such as cash payments, medicines or supplies a patient is asked to purchase from an outside pharmacy, or gifts given voluntarily to express gratitude. Within the same country, rates may vary over time or due to methodological differences (e.g. recall periods) [34]. Rates may also be affected by reticence, or respondents’ unwillingness to disclose having made an informal payment [38].

#### Ghost workers and absenteeism

**Figure 2.**
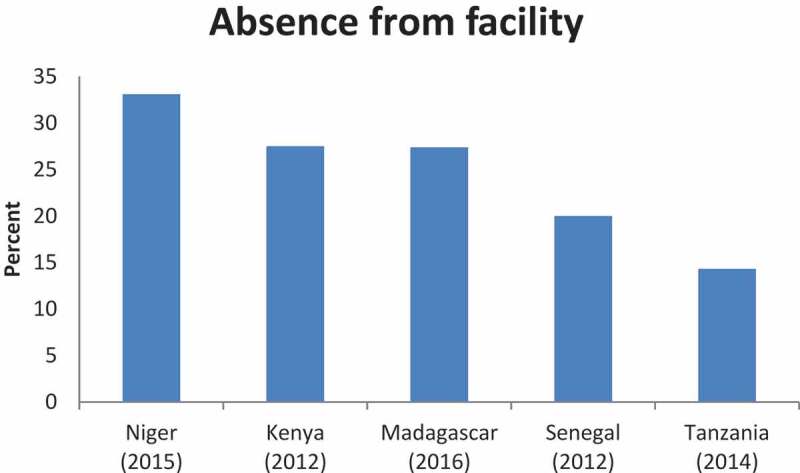
Health worker absence from health facility rates in 5 African countries, 2012–2015. Source: Service Delivery Indicator Survey, World Bank in partnership with the African Economic Research Consortium and the African Development Bank, www.sdindicators.org, Accessed 28 January 2018.

Another measure of corruption is the number of ghost workers, i.e. a person fraudulently added to the payroll who does not actually work. For example, a World Bank Public Expenditure study conducted in Honduras in 2001 found that 8.3% of general practitioners on payroll were ‘ghosts’ [39]. A related measure is unsanctioned absenteeism, where a worker is legitimately on the payroll but is chronically absent without approved reason [25]. In Honduras, unexcused absenteeism was 26% across all staff categories in the previously cited 2001 study, while a study conducted in 2004 found absenteeism in Bangladesh was 42% among physicians and 35% among other staff [39,40]. In Rwanda, a 2015 study found one-third of health workers in primary care facilities were absent [41]. World Bank Service Delivery Indicator Survey data from Africa in 2012–2016 show absent rates ranging from 14.3% of health facility staff in Tanzania, to 33.1% in Niger (Figure 2).

#### Dual practice and corruption risks

Clinicians who hold a salaried public sector job while maintaining a private practice are engaged in dual practice. Dual practice presents opportunities for corruption: for example, a physician may divert medicines, equipment, vehicles and fuel, funds, and patients from the public sector to her own private practice or ancillary services in which she has a financial interest [35,42–45]. Dual practice may reduce the availability and quality of services in the public sector, and exacerbate inequities in health worker distribution. It can also increase absenteeism and out-of-pocket or informal payments [44–46]. Surveys can estimate the extent and types of dual practice, and qualitative data collection may shed light on possibilities for abuse of power. This is a complex systems problem, and the risks of dual practice must be considered along with the potential positive consequence of enhancing health worker retention [46].

#### Reimbursement fraud and abuse

Insurance billing fraud refers to intentional deception or misrepresentation made with the knowledge that the deception could result in unauthorized benefits, while abuse is used to describe problematic behavior which is not clearly against the law or where certain elements of the fraud definition (such as knowing deception) are missing [47]. Organizations and governments use audit reports and statistical methods to try to estimate the scope of the problem. For example, a study published in JAMA and using data from 2011 estimated that $82 to $272 billion annually is lost to fraud and abuse in the U.S., or 3–10% of GDP [48]. Based on data collected from 33 organizations in 7 countries, Gee and Button estimate global average losses from healthcare fraud and abuse to be 6.19% of total health expenditure, or $455 billion in 2015 [49].

#### Substandard and falsified medical products

Substandard and falsified (SF) medical products enter markets in part due to regulatory failures connected to corruption. SF products cause unnecessary morbidity, mortality, and antimicrobial resistance [50,51]. An estimated 122,350 malaria deaths in children under five were associated with poor quality anti-malarial medicines, about 3.75% of all child deaths in 39 sub-Saharan African countries [52]. SFs are increasing in prevalence, especially in Africa [53]. A systematic review and meta-analysis of 96 studies published before November 2017 found that 13.6% of essential medicines tested in low- and middle-income countries (LMIC) failed quality analysis overall, with regional prevalence of 18.7% in Africa, and 13.7% in Asia [53]. A 2013 systematic review examined prevalence data from 15 studies of specific antimicrobials. The median prevalence of SF medicines was 28.5% (range 11–48%) [54]. A study of Ciprofloxacin treatment from 10 Latin American countries found that 7% of medicines were not good quality. Corruption in regulatory systems was a key predictor of poor quality drugs [55].

### Consequences: how corruption and lack of transparency and accountability undermine health

Corruption and lack of transparency and accountability undermine health by limiting equitable access to health services and financial protection [56,57]. In countries with greater corruption, citizens report being less satisfied with the quality of health services [58]. Pervasive corruption and lack of transparency also influence other health determinants such as access to clean water, sanitation, food, and housing [59–62].

Researchers estimate that 1.6% of world deaths in children, or 140,000 child deaths per year, could be indirectly attributed to corruption [63]. Countries with greater levels of corruption have higher rates of infant, child, and maternal mortality [64–67]. Public spending on health had a stronger effect in reducing child mortality in countries with lower levels of corruption and higher institutional capacity [68]. A 2010 study in 64 countries found that bribery was correlated with higher death rates for women giving birth, even after adjusting for per capita income and share of total spending on health [69]. Using 2000–2004 data from the World Health Survey, researchers determined that corruption was consistently associated with poor health among African adults in 20 countries [70].

Absenteeism can lead to significant negative health consequences for mothers and children. A study conducted using 2005–2007 longitudinal data from an antenatal clinic in Western Kenya found that women who were not tested during their first antenatal visit due to nurse absenteeism were 50 percentage points less likely to learn their HIV status during pregnancy [71]. Indicators of good governance, including control of corruption, are significantly associated with higher antiretroviral therapy (ART) and prevention of maternal to child transmission (PMTCT) coverage [72]. Corruption is associated with antibiotic resistance in Europe, possibly because corrupt governments provide less supervision of antibiotic use, especially in the private sector [73,74]. Finally, corruption may have an adverse effect on mental health: researchers found a strong link between individuals’ experience of corruption and self-reported anxiety [75]. The authors suggest that the sense of victimhood, uncertainty, unfairness, and ethical discomfort associated with corruption may drive anxiety, building the case for anti-corruption interventions as a way to increase health and well-being [75].

Informal payments are a barrier to accessing health care services (Table 2), causing greater harm for poor and rural populations [36,76,77]. A study of 33 African countries found that informal payments were concentrated among the poorest, indicating a regressive payment system in all but four countries (Côte d’Ivoire, Lesotho, Namibia and Swaziland) [36]. Using data from 2009–2012, researchers in Moldova highlighted the regressive nature of out-of-pocket payments (including informal payments). They observed that 29% of respondents in the poorest quintile could not afford medicines or services compared to only 5% in the highest quintile, and 22.3% of rural residents did not seek care due to financial reasons, compared to 6.2% of urban residents [78].

**Table 2.** Consequences and correlates of informal payments in the health sector.

Consequences of informal payments
Deters people from seeking care when needed.
Poses a financial burden on families, leading to higher levels of poverty and inequality.
Reinforces a two-tiered system, where people from low-income households seek care in less specialized facilities to avoid informal payments, while wealthy households have access to advanced and specialized treatment.
Prevents or delays health reforms, as individuals benefiting from informal payments (high-income households and healthcare providers) resist reform.
Undermines social justice in society and trust in the healthcare system, as people are forced to pay for care that should have been provided free of charge or was already paid for through official fees or premium payments.
Correlates of informal payments
**Household wealth**: being from a wealthier household is associated with a higher likelihood of informal payment because wealthier households have more resources to pay.
**Employed by government**: Working for the government is associated with a lower likelihood of informal payment: government workers have contacts and access to influence that can help them avoid having to pay.
**Healthcare quality**: Lower service quality in terms of long wait times, lack of medicines, absence of personnel, and disrespectful treatment are associated with higher likelihood of informal payment, as people seek to pay in order to be attended to by staff, or to jump a queue.
**Health status**: Worse self-assessed health status is associated with higher likelihood of informal payment, as people have to use services more frequently and have more concern about the impact on their health if they do not pay.
**Social network**: Having a friend, relative, or classmate who can ‘help when needed’ is associated with higher likelihood of informal payment, because the social network can be used to obtain resources to pay, and social norms influence individual behavior.
**Regional and country characteristics**: Differences in region and country political, economic, and social factors may affect the practice of informal payments; for example, more transparency and accountability in the healthcare system and public administration in general may reduce rates.

Source: Habibov and Cheung, 2017.

Audits reveal specific financial losses to corruption. For example, an audit of $17 million dollars in government Ebola spending in Sierra Leone found that one-third of money was not properly accounted for [26]. According to a statement issued by the International Federation of Red Cross and Red Crescent Societies, there were identified losses of 6 million Swiss Francs ($5.9 million USD) in Ebola funding due to embezzlement, fraudulent billing, over-inflated prices, and procurement corruption [79]. The Office of the Auditor General in Zambia uncovered embezzlement and unaccounted funds totaling $7.7 million, involving Ministry of Health employees who were organizing fake workshops and taking advances that were never repaid [80,81]. Auditors documented weaknesses in audit reports over several years, but no one acted on these findings, leaving administrative systems vulnerable to corruption.

### Frameworks for anti-corruption, transparency, and accountability

We identified six typologies and frameworks relevant to corruption in the health sector. Table 3 summarizes the purpose of the framework, and its constructs. This section also describes several models related to transparency and accountability.

**Table 3.** Frameworks and typologies of corruption, transparency, and accountability in the health sector.

Framework	Purpose	Constructs/definitions
EHFCN Waste Typology© European Healthcare Fraud and Corruption Network, 2014 www.ehfcn.org/what-is-fraud/ehfcn-waste-typology-matrix	To clarify anti-fraud definitions; avoid semantic confusion when exchanging information on counter fraud activity; and allow benchmarking	**Errors**: unjustly obtaining a benefit of any nature by unintentionally breaking a rule or a guideline **Abuses**: unjustly obtaining a benefit of any nature by knowingly stretching a rule or guideline or by taking advantage of an absence of rule or guideline **Fraud**: illegally obtaining a benefit of any nature by intentionally breaking a rule **Corruption**: illegally obtaining a benefit of any nature by abuse of power with third party involvement
European Union Corruption in the Healthcare Sector Typology, 2013 (updated 2017) https://ec.europa.eu/home-affairs/sites/homeaffairs/files/what-is-new/news/news/docs/20131219_study_on_corruption_in_the_healthcare_sector_en.pdf	To come to an analytically, practically and policy-wise meaningful grouping of corruption in health. To clarify various forms of corruption for a deeper analysis of the drivers and prevalence of corruption in health	**Bribery in medical service delivery**: Informal payments offered by patients or demanded by service providers. The 2017 update renamed this category “privileged access to medical services” also including use of privileged information **Procurement corruption**: Occurring throughout bidding cycle, involves bribes to individuals or institutions, collusion, favoritism, false invoicing, etc. **Improper marketing relations**: Problematic interactions between industry and providers or regulators (gifts, money, sponsorship, fees) which may bias decisions. Involves prescription influencing, undue promotion, and influence on market authorization and reimbursement of medicines/medical devices. **Misuse of high-level positions**: Regulatory state capture, trading in influence, conflicts of interest, favoritism and nepotism. Involves regulators, political parties, industry and providers. **Undue reimbursement claims**: Upcoding, reimbursement of unnecessary or non-delivered treatments. Involves payers and providers. **Fraud and embezzlement of medicines and medical devices**: Sale of public or prepaid medicines for private gain; sale of counterfeit medicines; use of publicly owned or financed devices or facilities for private gain. Involves providers. **Double practice**: The 2017 update considered risks associated with dual practice.
Typology of Individual and Institutional Corruption Sommersguter-Reichmann, et al., 2018, citing Thompson (2013) and Oliveira (2014)	To help determine what is to count as corrupt and to help prevent conduct already known to be corrupt	**Individual corruption**: When an institution or a public official receives a personal gain or benefit in exchange for promoting private interests (usually undeserved). The conduct does not serve the institution and involves a quid pro quo motive. **Institutional corruption**: When an institution or a public official receives a benefit while providing a service to the benefactor under conditions that undermine procedures that support the primary purposes of the institution.
Five Key Actors in the Health System, William D. Savedoff and Karen Hussmann, 2006	To identify possible types of corruption based on opportunities and interests that encourage corrupt behavior among the different categories of actors involved and the complexity of their multiple forms of interaction	**Government regulator**. Defines and approves norms for construction, equipment, medicines approval and control which can affected by state capture; may accept bribes to overlook compliance issues; inspectors may extort suppliers or providers. **Payer** e.g. social security, private or public health insurance. Affected by supplier influence on decision-makers (bribes, kickbacks related to procurement). May set negative incentives to save costs. **Drug & Equipment and Other suppliers**. May attempt to influence prescription and treatment practices, could engage in corruption in medicine and equipment procurement, procurement of facilities and ambulances. **Provider**. May engage in over-provision, overbilling, phantom patients, absenteeism, unnecessary treatment and prescriptions, demand informal payments **Patients**. May engage in fraud in beneficiary ID use, or understatement of income to obtain benefits
OECD framework of integrity violations in health care systems, Couffinhal and Frankowski, 2017	To link health care system actors to the main types of integrity violations they are involved in; to help organize categories of policy options to tackle integrity violations	**Actors** **Regulators** (ministry or dedicated agencies) **Payers** (entities that pool funds and finance care) **Suppliers and manufacturers** of medical goods and services **Providers** of medical goods and services **Individuals** (patients, tax-payers, or the insured) **Categories of integrity violation** **Integrity violations in health service delivery, payment and coverage** (denial of coverage, payroll tax evasion, informal payments, absenteeism, and over-billing). **Integrity violations in procurement and distribution** (bid-rigging, kickbacks, SF medicines). **Inappropriate business practices** (gifts and advantages to influence prescribing; corruption to influence regulation of private insurance market; bribes to obtain license or accreditation, systemic corruption).
Framework of Corruption in the Health Sector, Vian, 2008	To model the proximate causes and enabling factors that promote or impede corruption in the health sector	**Proximate causes** for individual corruption include opportunities to abuse power (gaps in control systems, excess discretion, etc.); pressures or incentives (which provide motivation to abuse), and rationalizations (how agents justify abuse of power). **Enabling factors** which allow individual or institutional corruption include monopoly (limiting choice or ability to exit a corrupt system); too much discretion (autonomous power to make decisions); lack of accountability; lack of transparency; weak citizen voice (participation of citizens in planning and monitoring government); and inadequate detection and enforcement.

EHFCN = European Healthcare Fraud and Corruption Network; SF = Substandard or Falsified; ID = Identification card Sources: EHFCN. EHFCN Waste Typology ©. 2014; www.ehfcn.org/what-is-fraud/ehfcn-waste-typology-matrix. Accessed 7 August 2018; Ecorys. *Study on Corruption in the Healthcare Sector*. HOME/2011/ISEC/PR/047-A2. October 2013. Luxembourg: Publications Office of the European Union; 2013; Ecorys. *Updated Study of Corruption in the Health Sector. Final Report*. Brussels: European Commission;2017; Sommersguter-Reichmann M, Wild C, Stepan A, Reichmann G, Fried A. Individual and Institutional Corruption in European and US Healthcare: Overview and Link of Various Corruption Typologies. *Applied Health Economics and Health Policy*. 2018;16(3):289–302; Savedoff WD and Hussmann K. Why are health systems prone to corruption? p. 4–16 in Transparency International, *Global Corruption Report 2016: Special Focus Corruption and Health*, London: Pluto Press. 2016; Vian T. Review of corruption in the health sector: theory, methods and interventions. *Health Policy Plan*. 2008;23(2):83–94; Couffinhal A, Frankowski A. Wasting with intention: Fraud, abuse, corruption and other integrity violations in the health sector. In: OECD, ed. Tackling Wasteful Spending on Health. Paris: OECD Publishing; 2017:265–301.

#### Corruption frameworks

Two of the typologies (EHFCN Waste Typology© and Corruption in the Health Sector Typology) focus on types of wrongdoing in the European health sector context [35,82,83]. Other frameworks link specific actors to types of corruption or integrity violations (Five Key Actors in the Health System model, OECD Integrity Violations Framework) [84,85]. The fifth framework (Typology of Individual and Institutional Corruption) is used to distinguish corruption that involves individual abuse of power, compared to corruption where institutions engage in activities that promote ends which are not aligned with their primary purpose [5,86,87].

The sixth framework, Vian’s 2008 framework, synthesizes earlier work by corruption researchers including the fraud triangle theory [88] and Klitgaard’s heuristic model for anti-corruption [89] to model factors influencing corruption in the health sector [16]. These include pressures, opportunities, and rationalizations. Pressures contribute to corruption risk in several ways. For example, Hernández-Aguado and Chile-Rosell (2018) observed that health policies captured by interested actors may shift the policy-making process away from the public interest towards narrow private interests [90]. The authors identified pressures applied by parliamentarians to favor certain pharmaceutical companies, and described economic offers to join companies after leaving government (‘revolving door corruption’) and bribes or gifts as creating pressures which result in corrupt decisions [90]. Pressures can also be financial or structural such as low wages and benefits, inadequately equipped health centers, and lack of positive reinforcement to do what is right [43].

Opportunities for corruption include monopoly (which limits the choice available to citizens who are forced to interact with corrupt agents), too much discretion, limited accountability for performance, lack of transparency, weak citizen voice (participation by citizens in planning and monitoring government services), and failures in detection and enforcement to curb corruption.

#### Transparency frameworks

Public sector management experts distinguish between two types of transparency: access to information by recipients (event transparency) and administrative processes which are accessible, simple, and comprehensible (process transparency) [91]. Organizations need transparency in four directions. Upward transparency allows a supervisor to observe the conduct or results produced by a subordinate. Downward transparency allows subordinates to observe the conduct/results of supervisors. Outward transparency refers to when those inside an organization can see what is happening outside an organization (e.g. benchmarking). Inward transparency allows those outside an organization to see what is going on within [91].

Open public meetings is a mode to increase transparency [15]. Meetings provide an opportunity for government to share information and solicit citizen input, and for citizens to voice concerns and demand a response from officials. Piotrowski and Borry (2010) have created a transparency framework applicable to open public meetings at the local government level including necessary aspects such as notice and agenda, minutes, rules about holding closed sessions, public comment, and documentation [15].

In addition to modeling modes of transparency (like public meetings), stakeholders have adapted transparency models to topics such as clinical trials [92]. Clinical trial transparency addresses the problem that researchers sometimes avoid publishing disappointing trial results [93]. This practice can threaten the validity of clinical research literature and possibly affect the quality and safety of treatments [94]. The clinical trial transparency model promotes five standards: 1) trial registration (trials registered before they start); 2) summary results posting (key results made public within 12 months; 3) full trial reports (detailed findings proactively disclosed); 4) academic publication (trial results published with peer review); and 5) individual participant data sharing [92].

**Figure 3.**
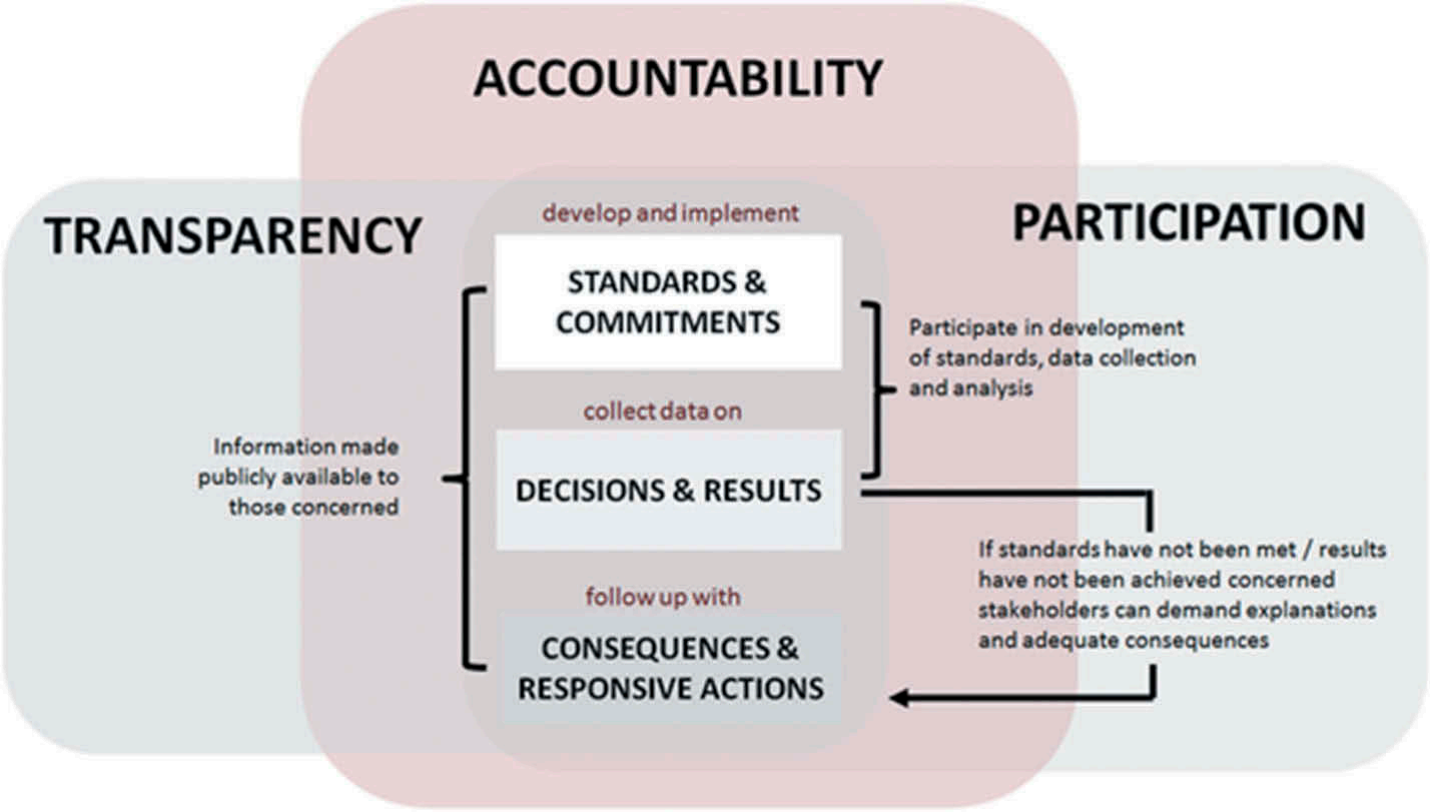
How transparency and participation enable accountability. Source: Adapted from Paschke, Dimancesco, Vian, et al. 2018.

Paschke and co-authors present a model (Figure 3) to show how transparency, combined with participation, can enable accountability. Information on standards and commitments, decisions and results, and responsive actions taken by government are used by citizens and oversight bodies to demand explanations (answerability) and consequences (sanctions) [10].

#### Accountability frameworks

Brinkerhoff (2004) argues that answerability, or the obligation of public officials to provide information on actions taken and to justify these actions to oversight actors, is the essence of accountability. Justification of actions involves a bi-directional exchange of information between accountable actors and those in positions of oversight [95]. Brinkerhoff’s conceptual model of accountability outlines three types: financial, performance, and political. Financial accountability assures proper allocation and use of resources through budget/accounting controls and participatory budget processes. Performance accountability examines the degree to which outputs align with pre-determined performance targets. Political/democratic accountability monitors the degree to which governments and institutions deliver on promises, act in the best interest of citizens, and respond effectively to societal needs [95]. To disrupt the cycle of abuse, accountability systems must identify responsible actors and determine patterns of interaction, and develop the capacity of actors to support accountability (promote answerability, impose and enforce sanctions) [95]. In 2014, Brinkerhoff and Bossert expanded on the idea of shared accountability that strategically engages non-governmental actors. This revised model identifies three categories of actors – citizens/clients, the state, and service providers [96]. A primary limitation of this model is its assumption of equal power and influence: in fact, providers and state actors generally tend to have more power than clients/citizens [96–98].

Jonathan Fox (2015) examines tactical and strategic approaches to social accountability involving citizens and civil society organizations. He proposes a ‘sandwich strategy’ framework, focused on the mutual empowerment of state actors (reformists with power over policy implementation who create opportunities for citizen voice) and citizens (engaged in public interest advocacy and collective action). The opportunity for change can arise from pro-accountability forces in the society or state; the key to success is the ability and willingness of counterparts in the other domain to support and empower others to act. Practical actions for improving accountability include providing user-centered information; establishing communication channels through which citizens exercise voice in a collective manner and state actors can respond to citizen demands; addressing past accountability problems (i.e. citizen fear, lack of enforcement of sanctions); and enhancing the capacity of states to demand accountability from providers and impose sanctions.

### Anti-corruption approaches

Strategies for anti-corruption should align with national anti-corruption institutions and strategies, and need to consider both prevention and enforcement [99,100]. Recent analyses have reviewed evidence of the links between ACTA initiatives, and health outcomes [101], public financial management and health systems performance [102], and technology and good governance [103]. Additionally, a Cochrane Review assessed evidence of health sector anti-corruption interventions [104]. Although the authors noted a paucity of rigorous effectiveness research on this topic, they found some evidence in support of four strategies. These include: 1) fraud control and independent complaint mechanisms (evidence from the US and India); 2) guidelines regulating physician-industry interactions (evidence from Europe and US); 3) internal controls, rules and procedures to strengthen financial and accounting systems in health centers (evidence from US); and 4) increased transparency and reduced incentives for informal payments (evidence from Kyrgyzstan health reforms) [104].

We briefly mention some intervention strategies and highlight areas where stakeholders are working to increase transparency, strengthen accountability, and fight corruption. Categories include corruption risk assessment, transparency interventions, complaint mechanisms, audit, SF medicines, and informal payments.

#### Risk assessment

Hussmann (2011) has catalogued many tools available to identify, track and measure health sector corruption (Table 4) [105]. Health sector corruption risk assessments have been conducted by UNDP, Council of Europe, USAID and others [106,107]. Separate assessment tools have analyzed financial management systems for compliance with anti-corruption measures [108,109] and reviewed health laws for anti-corruption provisions [110]. In the MENA region, UNDP is working on corruption risk mapping as part of long-term engagement based on prevention, risk management, and multi-stakeholder engagement.

**Table 4.** Key tools to identify, track and measure corruption risks.

**General Tools**	
Cross-cutting	Political economy analysis; vulnerability to corruption assessments; value chain analysis; sectoral accountability assessment; value for money audits; analysis of governance in health care systems
**Budget and Resource Management Tools**	
Budget process	Public expenditure and financial accountability indicators (PEFA); focus groups and interviews with public officials, recipient institutions, and civil society
Payroll leakages	Public expenditure tracking surveys and reviews (PETS, PERS); household surveys; focus groups with public officials and health workers
In-kind leakages	PETS; quantitative service delivery surveys (QSDS); facility surveys; focus groups with public officials, recipient institutions, and health workers
Pharmaceuticals	GGM Programme; International Drug Price Indicator Guide; internet-based drug procurement price databases
**Individual Provider Tools**	
Job purchasing	Official administrative records combined with facility surveys; interviews with public officials and former officials; governance and anti-corruption country diagnostic surveys
Health worker absenteeism	QSDS; surprise visits; direct observation; facility records; focus groups or interviews with facility heads and patients
**Informal Payments tools**	
Informal payments	Household surveys (World Bank Living Standards Measurement Survey, Demographic and Health Survey); facility exit surveys and score cards; focus groups and interviews with patients, providers, and staff; Governance and Anti-Corruption Country Diagnostic surveys
**Corruption perceptions and experience**	
Perceptions	World Bank Governance Indicators, TI Corruption Perception Index; World Bank Governance and Anti-Corruption Country Diagnostic surveys; national level perception surveys by CSO and others
Experiences	AfroBarometer; LatinBarometer; EuroBarometer; TI Global Corruption Barometer; National experience based surveys; patient satisfaction surveys; report (score) cards; focus groups

Source: Hussman, 2011.

#### Transparency interventions

WHO has guided two major advances to promote transparency in order to fight corruption, including the Medicines Transparency Alliance (MeTA), a multi-stakeholder initiative implemented in seven countries between 2008 and 2015, and the Good Governance in Medicines (GGM) Programme, which included a transparency assessment tool for the pharmaceutical sector. These initiatives, described in detail elsewhere, are briefly summarized below [97,111,112]. A third major category of transparency interventions is community monitoring, report cards, and social audits.

- Medicines Transparency Alliance (MeTA). The Medicines Transparency Alliance (MeTA) is a multi-stakeholder voluntary partnership to bring together government officials, NGOs, and pharmaceutical companies to increase trust and pressure for greater accountability and evidence-based policy-making. Data disclosure surveys conducted in each of the seven MeTA countries assessed public disclosure of information on medicines policies, practices, and availability and price indicators. An evaluation found that MeTA achieved its goal of creating the multi-stakeholder shared space in which government, civil society, and private sector players could have a voice in policy making [112]. In Jordan, MeTA successfully advocated for a government-run medicine policy unit to build capacity for collecting and analyzing access data. In Peru, MeTA created an observatory with government-mandated reporting of medicine prices [112]. In some countries, power imbalances seemed evident: CSOs did not have the same power to engage in policy dialogue as other stakeholders [97].
- Good Governance in Medicines Programme. WHO’s Medicines Strategy 2004–2007 highlighted corruption as a priority. In response, WHO launched the Good Governance in Medicines (GGM) Programme, involving 38 countries in transparency assessment and development/implementation of a national pharmaceutical governance framework [10]. A 2013 evaluation report found the program had promoted revised pharmaceutical laws and regulations, leading to improved transparency and more active management of conflicts of interest [113]. Further evaluation research is needed to assess how these tactics affect policy and procedures to control corruption in the pharmaceutical sector.

#### Community monitoring, report cards, and social audit

A recent review cites evidence from Malawi, Uganda, and Tajikistan supporting the effectiveness of report cards and community monitoring [101]. The Transparency for Development project used scorecards in their citizen-led accountability programs in Tanzania and Indonesia [114]. The program allowed citizens to develop plans to deal with the specific problems facing their community, and choose contextually-appropriate actions to further accountability [114]. However, this program did not have a statistically significant impact on use or content of maternal and newborn health services [115]. The authors believe this is because the causal pathways to improving services were long and complicated, and it was hard for citizens to translate their plans into actions [115]. UNESCO International Institute for Educational Planning concluded that creating report cards and sharing performance data must be accompanied by political action to modify power imbalances to ensure the data are used effectively, and that ‘malpractice is addressed with clear consequence’ (Table 5). The International Budget Partnership promotes social audit as a technique to monitor service delivery and expenditures [116,117].

**Table 5.** Recommendations to use report cards to promote transparency and accountability.

(1) Create legal provisions for disclosure of facility data
(2) Formulate a clear theory of change that makes the link between data and accountability
(3) Consider power imbalances and cultural constraints when designing an open data policy
(4) Select data that are critical to monitoring financial, management, or clinical accountability
(5) Prioritize data that are relevant for patients and citizens to encourage them to take part in health facility accountability efforts
(6) Design mechanisms enabling fair comparisons between facilities
(7) Simplify the presentation of data while maintaining their technical accuracy
(8) Create a range of avenues (both online and offline) for citizens to access data
(9) Train facility management committees, health workers, patient interest groups, and selected community groups on how data can be used to demand accountability
(10) Introduce a legal grievance redressal mechanism for patients and communities

Source: Adapted from http://www.iiep.unesco.org/en/10-ways-promote-transparency-and-accountability-education-4307.

#### Whistleblowing and complaint mechanisms

Internal whistleblowing, and complaint mechanisms for patients, are important ACTA tools [1]. They allow organizations to investigate or refer alleged corruption incidents, identify and discipline or educate careless or incompetent staff, and revise unworkable official procedures that may be leading to abuses or unaccountable actions. These mechanisms can be set up at the project, organization, sectoral, or country level. Protection for whistleblowers is essential.

In Karnataka state, India, a Health Vigilance Officer (part of the Karnataka Lokayukta, a public complaints agency) visited 202 administrative units between 2001 and 2006, handling 100–200 complaints per visit [118]. Over 800 complaints were serious enough to refer to police. However, some cases were not prosecuted because police lacked investigative capacity. Other prosecution efforts revealed conflicts in State versus Federal authority that were difficult to resolve. Committed leadership and willingness of citizens to complain was not enough to overcome the legislative and justice system constraints which limited the extent to which officials could be held accountable [119]. In Albania, the government’s anti-corruption complaint online portal received 1,605 health-related complaints (10% of total) between 2013–2017 [120]. About 75% were determined not to be corruption-related, including complaints about long lines and poor quality services. In addition, with support from a World Bank loan, the Prime Minister’s Office implemented a citizen feedback text messaging intervention. From October 2016 until August 2017, the top three issues identified in hospitals were poor conditions, lack of drugs, and unqualified staff. Citizens regarded the program favorably because they felt listened to. Such a program may help to deter corruption because public officials know that the text messages are being sent, and that citizens may report wrong-doing [120]. Yet, complaint mechanisms can only work if there is adequate staffing, resources, and ability to handle the complaints raised. Governments must check on whether health officials have addressed complaints.

#### Audit

Audit can be a powerful tool to identify malfeasance. External audit of the GAVI program (formerly the Global Alliance for Vaccines and Immunizations) in Cameroon covering the period 2008 to 2010 and first quarter of 2011 revealed $3.1 million in misspent funds, including fraud [108]. Audit reports can trigger prosecutions, and the threat of regular or surprise audits may have a deterrent effect on corruption [102]. Researchers have studied the link between corruption and effective internal audit function in Ghana, a decade after a 2003 reform to strengthen the Internal Audit Agency [121]. They found that size and independence of the internal audit department were associated with effectiveness of the internal audit in reducing corruption. They conclude that full implementation of internal audit regulations and laws, and assurance of the independence of the internal audit function, can help fight administrative corruption [121].

Supreme Audit Institutions (SAI) are government agencies responsible for oversight of public expenditures. They may have different names, models or mandates in different countries, but are defined by their role of external audit on government expenditure and performance, as part of a larger national integrity system. A recent literature review found no studies on the role of SAI and the health sector [102]. The Albania Transparency in Health Engagement project (2017–2019) worked with the Albanian SAI to increase the number and quality of performance audits in the health sector [122]. The project is building capacity of auditors understand the complex referral and treatment systems in the health sector, and to effectively work with civil society to document problems. There is a need for more research to document a) the quality of internal and external audit capacity in the health sector, b) the effect of audit on corruption in the health sector.

#### Systems-level approaches to manage substandard and falsified medicines

A 2016 systematic review examined international experience with strategies to detect and prevent SF medicines [51]. They recommended a systems-level approach to protect the supply chain. Given the global trade in pharmaceuticals and presence of organized crime networks, international collaboration is essential for detection and successful prosecution. Examples of supranational organizations supporting such efforts include the Pharmaceutical Security Institute Counterfeit Incident System (PSI CIS), the WHO Medical Product Alert System, and VigiBase, a global database of individual case safety reports (adverse events, product complaints etc.). The WHO Member State mechanism on substandard and falsified medical products produces a workplan with activities related to training, expanding focal point networks, and improving member states’ understanding of detection technology and ‘track-and-trace’ models [123]. Participation in systems like these requires a well-functioning national medicine regulatory agency with adequate capacity, something that is still lacking in many Sub-Saharan African countries [51]. France, Norway, and the Netherlands have increased the terms of incarceration for people convicted of falsifying medicines, and are raising consumer awareness to help them identify fake medicines, thus reducing the potential financial benefits of selling fake medicines [51].

#### Standardized monitoring of informal payments

Many studies have measured informal payments for health services; however, methodological differences complicate data comparisons. Khodamoradi et al. (2017) noted differences in how informal payments are defined, locations (e.g. hospital or primary care), sample size, sample selection and data collection, recall period, and how affordability measures are calculated [22]. Some studies include medicines bought outside the facility as informal payments, while others do not [21], and some studies try to separate gifts from other types of informal payment [124]. Research designs with probabilistic sampling (e.g. random or stratified) may provide more generalizable estimates compared to research conducted with convenience samples, and it is important to employ methods adapted to the potentially sensitive issues where people may not feel comfortable admitting they made payments [125]. Khodamoradi et al. have proposed creating a universal research map for standardized definitions, language, and methods, and recommended a global study to assess informal payments in all countries using such a tool.

#### Addressing under-resourced health systems

Pressure to perform without having adequate funding, human resources, or institutional capacity is a barrier to achieving health goals. Resource limitations can increase risk for corruption, in addition to corruption making resources less available [126]. Informal payments were characterized by one research team as ‘a saving mechanism for those wanting to escape the limitations of a continuously decaying health system’ [127]. The U.S. Veterans Administration faced a scandal in 2014 that also illustrates this connection [128,129]. There was a mismatch between performance goals and adequate funding to reach those goals. As a result, employees felt pressured to falsify data and hide actual performance failures [130].

## Potential areas for research

To document and compare corruption risks and patterns, researchers and international organizations should develop guidance on risk assessment, including how to mine existing databases or collect primary data (interview guides, transparency assessment tools, methods for assessing problems like absenteeism and ghost workers). WHO could adopt standardized methods for collecting and reporting indicators on informal payments, to address the wide variation and lack of comparability in methods currently being used [21,22,34,124]. Measurement and assessment tools need to be validated.

Specific interventions which merit further study include fraud control and data mining; transparency policies; whistleblowing and complaint mechanisms; community monitoring; management control tools and health sector audit strengthening; conflict of interest management; regulatory reform to incentivize payers and provider organizations to control corruption.

For each area of intervention, several research questions could be relevant. First, research requires a clearly defined intervention, strategy, and expected outcomes. For example, what are the essential features or components of a staff whistleblowing program or patient complaint mechanism? This work will need to draw from theory and practice in specific settings and types of health organizations. It requires thinking through the theory of change for each intervention, and considering how we will measure outcomes. It may be possible to develop a core model, but researchers will also need to consider how contextual factors will influence the model. A second area of research is effectiveness studies, to determine if interventions work in practice. Finally, research is needed to assess how strategies for implementation affect the adoption, use, and impact of interventions to control corruption risk. Implementation science researchers could examine why some institutions adopt management control tools while others do not. Implementation science research could also address the impact of attitudes, skills, knowledge, or the enabling environment on scaling up interventions.

## Conclusions

This study documented concepts, frameworks, and approaches to identify anti-corruption strategies and to address the consequences of corruption in health systems. We found six recent frameworks that may help practitioners understand and categorize corruption risk and elements to consider as levers for anti-corruption interventions. A growing body of literature suggests that corruption endangers progress in achieving better health, economic growth and development. Corruption is a barrier to achieving UHC, and requires preventive actions and risk mitigation. Yet, our review also finds that research to design, implement, and evaluate evidence-based policy and management interventions is lacking. The global community should focus greater attention and resources to strengthen health systems to control corruption and to promote transparency and accountability throughout the health sector.

## Paper context

Ten years ago, the author published a review on corruption in the health sector that is widely cited. This article updates the literature published from 2008 to 2018, and documents the context in which corruption in the health sector is occurring, frameworks for understanding corruption drivers and consequences, and approaches to prevent and curb corruption through anti-corruption measures, transparency, and accountability. Understanding context, frameworks, and approaches is important to fight this threat to our health goals.

## Appendix A. Summary of Citations from Systematic Review of Corruption and Health Frameworks, Models and Theory

**Table:**

	Authors	Year	Title	Journal	Theoretical constructs/relevance
1	Sommersguter-Reichmann, M; Wild, C; Stepan, A et al.	2018	Individual and institutional corruption in European and US healthcare: overview and link of various corruption typologies	Applied Health Economics and Health Policy	Compared European Union typology with EHFCN and Thompson (individual vs. institutional corruption) frameworks
2	Shi, JW; Liu, R; Jiang, H et al.	2018	Moving towards a better path? A mixed-method examination of China’s reforms to remedy medical corruption from pharmaceutical firms	BMJ Open	Describes and critiques anti-corruption initiatives implemented in China to reduce pharmaceutial sector corruption
3	Serneels, P; Lievens, T	2018	Microeconomic institutions and personnel economics for health care delivery: a formal exploration of what matters to health workers in Rwanda	Human Resources for Health	Analyses and concepts related to absenteeism
4	De Mendonca, HF; Baca, AC	2018	Relevance of corruption on the effect of public health expenditure and taxation on economic growth	Applied Economic Letters	Impact of corruption on allocation of resources, including public health expenditures
5	Mfutso-Bengo, J; Kalanga, N; Mfutso-Bengo, EM	2017	Proposing the LEGS framework to complement the WHO building blocks for strengthening health systems: One needs a LEG to run an ethical, resilient system for implementing health rights	Malawi Medical Journal	Proposes a leadership, ethics, governance, and systems (LEGS) framework to complement the WHO health systems building blocks, especially in countries with high levels of corruption
6	Mladovsky, P; Ba, M	2017	Removing user fees for health services: A multi-epistemological perspective on access inequities in Senegal	Social Science & Medicine	Examines inequities in access to free health care. Linked to corruption, patronage, information dissemination, social networks
7	Hunter, BM; Murray, SF	2017	Demand-side financing for maternal and newborn health: what do we know about factors that affect implementation of cash transfers and voucher programmes?	BMC Pregnancy and Childbirth	Cash transfer and voucher programs may advance anti-corruption goals. Fraud detection systems may reduce corruption but could cause delays in financial flows.
8	Zuhaira, MAM; Tian, YZ	2017	The effect of religious beliefs, participation and values on corruption: survey evidence from Iraq	International Journal of Advanced Computer Science and Applications	Religious beliefs may reduce corruption through social pressure and raising of standards.
9	Pyone, T; Smith, H; van den Broek, N	2017	Frameworks to assess health systems governance: a systematic review	Health Policy & Planning	Describes governance frameworks from different disciplines with focus on accountability, and linkages to anti-corruption.
10	Stephens, T; Mezei, A; O’Hara, NN et al.	2017	When surgical resources are severely constrained, who receives care? Determinants of access to orthopaedic trauma surgery in Uganda	World Journal of Surgery	Identified social capital (social connections) and financial leverage (offering money) as determinants of access to surgical care
11	Stepurko, T; Pavlova, M; Gryga, I et al.	2017	Patterns of informal patient payments in Bulgaria, Hungary and Ukraine: a comparison across countries, years and type of services	Health Policy & Planning	Describes factors driving informal payments and possible anti-corruption measures
12	Ronnerstrand, B; Lapuente, V	2017	Corruption and use of antibiotics in regions of Europe	Health Policy	Associates corruption with variation in antibiotic consumption
13	van Deurzen, I	2017	And justice for all: examining corruption as a contextual source of mental illness	Social Science & Medicine	Discusses impact on corruption on mental health indicators
14	Rispel, LC; de Jager, P; Fonn, S	2016	Exploring corruption in the South African health sector	Health Policy & Planning	Lack of internal controls and enforcement linked to corruption, with impact on patient care and health worker morale
15	Gillanders, R	2016	Corruption and anxiety in Sub-Saharan Africa	Economics of Governance	Strong correlation between corruption and anxiety
16	Gaitonde, R; Oxman, AD; Okebukola, PO et al.	2016	Interventions to reduce corruption in the health sector	Cochrane Database of Systematic Reviews	Found few high quality studies; some suggestion that better internal controls, community monitoring may work
17	Factor, R; Kang, M	2015	Corruption and population health outcomes: an analysis of data from 133 countries using structural equation modeling	International Journal of Public Health	Constructs model to explain antecedents of corruption and effects on health outcomes. Corruption has strong effect on health outcomes, controlling for other factors.
18	Kohler, JC; Mitsakakis, N; Saadat, F et al.	2015	Does pharmaceutical pricing transparency matter? examining Brazil’s public procurement system	Globalization and Health	Shows that transparency alone didn’t work to lower prices
19	Pinzon-Florez, CE; Fernandez-Nino, JA; Ruiz-Rodriguez, M et al.	2015	Determinants of performance of health systems concerning maternal and child health: a global approach	PLOS One	Provides evidence that corruption is correlated with maternal mortality. Having more corrupt government was significant risk determinant. This relationship is strongest in African region.
20	Collignon, P; Athukorala, PC; Senanayake, S et al.	2015	Antimicrobial resistance: the major contribution of poor governance and corruption to this growing problem	PLOS One	Presents evidence that corruption is correlated with antibiotic resistance in Europe
21	Pinzon-Rondon, AM; Attaran, A; Botero, JC et al.	2015	Association of rule of law and health outcomes: an ecological study	BMJ Open	Associates the rule of law, including absence of corruption, with lower infant mortality rates
22	Lin, RT; Chien, LC; Chen, YM et al.	2014	Governance matters: an ecological association between governance and child mortality	International Health	Associates World Bank governance indicators (incl. control of corruption) with lower under 5 mortality
23	Man, WYN; Worth, H; Kelly, A et al.	2014	Is endemic political corruption hampering provision of ART and PMTCT in developing countries?	Journal of the International AIDS Society	Shows that political governance indicators are associated with levels of ART and PMTCT coverage
24	Siverson, RM; Johnson, RAI	2014	Politics and parasites: the contribution of corruption to human misery	International Studies Quarterly	Shows corruption has deleterious effect on health outcomes measured in DALYs, but not in Sub-Saharan Africa because effects of AIDS and malaria are stronger
25	Kohler, JC; Mackey, TK; Ovtcharenko, N	2014	Why the MDGs need good governance in pharmaceutical systems to promote global health	BMC Public Health	Summarizes several anti-corruption interventions to increase transparency and accountability in pharmaceutical systems
26	Nikoloski, Z; Mossialos, E	2013	Corruption, inequality and population perception of healthcare quality in Europe	BMC Health Services Research	Finds corruption is correlated with lower perceived quality of care
27	Hanf, M; Nacher, M; Guihenneuc, C et al.	2013	Global determinants of mortality in under 5s: 10 year worldwide longitudinal study	BMJ	Shows correlation of corruption and health outcomes
28	Wafula, F; Molyneux, C; Mackintosh, M et al.	2013	Protecting the public or setting the bar too high? Understanding the causes and consequences of regulatory actions of front-line regulators and specialized drug shop operators in Kenya	Social Science & Medicine	Identifies corruption between inspectors and the drug shops; distinguishes bribes by type; Analyzes organizational influences on this type of corruption (chain of command, reporting rules, incentives)
29	Jorgensen, PD	2013	Pharmaceuticals, political money, and public policy: a theoretical and empirical agenda	Journal of Law, Medicine & Ethics	Proposes a theoretical model of ‘dependence corruption’ (also called institutional corruption)
30	Cosgrove, L; Wheeler, EE	2013	Drug firms, the codification of diagnostic categories, and bias in clinical guidelines	Journal of Law, Medicine & Ethics	Provides example of dependence corruption or institutional corruption in clinical guideline setting
31	Ntayi, JM; Ngoboka, P; Kakooza, CS	2013	Moral schemas and corruption in Ugandan public procurement	Journal of Business Ethics	Describes types of procurement-related corruption and the justifications of staff for engaging in corruption
32	Mackey, TK; Liang, BA	2012	Combating healthcare corruption and fraud with improved global health governance	BMC International Health and Human Rights	Highlights how corruption can cross geo-political borders and affect global health outcomes such as response to epidemics, counterfeit medicines
33	Malqvist, M; Dinh, TPH; Thomsen, S	2012	Causes and determinants of inequity in maternal and child health in Vietnam	BMC Public Health	Provides a general description of corruption (informal payments) as a social determinant of inequities
34	Olken, BA; Pande, R	2012	Corruption in developing countries	Annual Review of Economics, Vol. 4	Analyzes absenteeism; provides some overall statistics about measuring corruption
35	Sur, H; Cekin, MD	2012	Ethical Conduct in Health Services in Turkey	Turkish Studies	Discusses dual practice and physician-pharma interactions
36	Peixoto, SGD; Rocha, FF; Nishijima, M et al.	2012	Decentralization and corruption: evidence from primary health-care programmes	Applied Economic Letters	Examined whether decentralization was associated with corruption in municipal health budgets in Brazil. Found no association
37	Hanf, M; Van-Melle, A; Fraisse, F et al.	2011	Corruption kills: estimating the global impact of corruption on children deaths	PLOS One	Measures the health consequences of corruption, estimating 140,000 child deaths per year indirectly associated with corruption
38	Huss, R; Green, A; Sudarshan, H et al.	2011	Good governance and corruption in the health sector: lessons from the Karnataka experience	Health Policy & Planning	Summarizes experience of a state government Vigilance (Ethics) Office and complaint mechanism to curb corruption in India
39	Muldoon, KA; Galway, LP; Nakajima, M	2011	Health system determinants of infant, child and maternal mortality: A cross-sectional study of UN member countries	Globalization and Health	Having a less corrupt government is associated with lower infant and child mortality, after controlling for other factors
40	Guinness, L	2011	What can transaction costs tell us about governance in the delivery of large scale HIV prevention programmes in southern India?	Social Science & Medicine	Analyzes impact of contracting out as anti-corruption measure; if using contract management agency, can reduce corruption by overcoming information problems
41	Dieleman, M; Shaw, DMP; Zwanikken, P	2011	Improving the implementation of health workforce policies through governance: a review of case studies	Human Resources for Health	Governance oversight promotes anti-corruption goals, accountability, and transparency; political interference, informal power relations, and favouritism distort regulatory systems and enable corruption
42	Chereches, R; Ungureanu, M; Rus, I et al.	2011	Informal payments in the health care system – research, media and policy	Transylvanian Review of Administrative Sciences	Finds regulatory action against informal payments is lacking, despite ubiquity of practice
43	Barr, A; Lindelow, M; Serneels, P	2009	Corruption in public service delivery: An experimental analysis	Journal of Economic Behavior & Organization	Monitoring works when monitors are elected by service recipients, and likely to be observed; paying higher wages had mixed effect; emphasizing professional norms had positive impact on younger service providers; exposure to corruption erodes social norms
44	Radin, D	2009	Too ill to find the cure? Corruption, institutions, and health care sector performance in the new democracies of Central and Eastern Europe and Former Soviet Union	East European Politics and Societies	Examined correlation between corruption and cancer mortality, with informal payments as mediating factor
45	Sakyi, EK	2008	A retrospective content analysis of studies on factors constraining the implementation of health sector reform in Ghana	International Journal of Health Planning and Management	Desribes patronage networks in Ghana and the many different forms of corruption including those related to dual job holding
46	Vian, T	2008	Review of corruption in the health sector: theory, methods and interventions	Health Policy & Planning	Provides framework of factors influencing corruption, describes consequences of corruption and anti-corruption approaches

## Appendix B.

- **List of Attendees at the WHO Technical meetings on Anti-corruption, Transparency, and Accountability, November 2017**
- **List of Attendees at the WHO Technical meetings on Anti-corruption, Transparency, and Accountability, March 2018**
- **References from the Background Paper for a WHO Technical Meeting on Anti-corruption, Transparency, and Accountability shared with participants in Geneva, Switzerland, March 22–23, 2018.**

**Attendees, November 2017 meeting**

***Accountability and Transparency Experts***

Dina BALABANOVA, Associate Professor, London School of Hygiene and Tropical Medicine (LSHTM)

Sebastian BAUHOFF, Research Fellow, Center for Global Development (CGDEV)

Anouar BEN KHELIFA, Investigations Specialist, United Nations Development Fund (UNDP)

Agnès COUFFINHAL, Senior Economist, Organisation for Economic Co-operation and Development

Arkan EL-SEBLANI, Regional Manager, UNDP Arab States, Anti-Corruption

Sarah HARRIS STEINGRÜBER, Programme Manager, Pharmaceuticals & Health Care Programme, Transparency International UK

Mostafa HUNTER, Consultant, UNDP Center for Transparency

Eleanor HUTCHINSON, Assistant Professor in Medical Anthropology, LSHTM

Twesiime Monica KIRYA, Senior Advisor, U4 Anti-Corruption Resource Center at the Christian Michelsen Institute

Roxanne OROXOM, Research Associate, CGDEV

Esther SAVILLE, Social Development Advisor, Transparency, Accountability and Politics Group in the Governance, Open Societies and Anti-Corruption Department, Department for International Development (DFID), UK

Taryn VIAN, Clinical Professor of Global Health, Boston University School of Public Health

Paul VINCKE, Managing Director, European Healthcare Fraud and Corruption Network

Aneta WIERZYNSKA, Senior Advisor, Anticorruption and Impact, Ethics Office, The Global Fund to Fight AIDS, Tuberculosis and Malaria

***WHO***

Agnès SOUCAT, Director, HGF

Hala ABOU TALEB, Technical Officer, EM/PHP

Marie-Charlotte BOUESSEAU, Advisor, SDS

David CLARKE, Health Systems Advisor (Legal), HGS

Ibadat DHILLON, Technical Officer, HWF

Deirdre DIMANCESCO, Technical Officer, EMP

Matthew JOWETT, Senior Health Financing Specialist, HEF

Theadora Swift KOLLER, Technical Officer for Equity, GER

Clotilde PETRIAT, Volunteer, HGS

Dheepa RAJAN, Technical Officer, HGS

Bruce TSAI, Intern, HGS

**Attendees, March 2018 meeting**

***Accountability and Transparency Experts***

Sebastian BAUHOFF, Research Fellow, Center for Global Development (CGDEV), Washington DC USA

Samar ELSAYED, Health Governance Consultant, Healthcare Governance and Transparency Association (HeGTA)

Frank FEELEY, Associate Professor, Department of Global Health, Boston University School of Public Health, Boston MA USA

Mennatallah Mohamed Hatem ELAYADI, Health Governance Consultant, Healthcare Governance and Transparency Association (HeGTA)

Mostafa HUNTER, Senior Advisor on Health, UNDP-ACIAC

Eleanor HUTCHINSON, Assistant Professor, Anthropology and Health Systems, LSHTM - London School of Hygiene and Tropical Medicine, London, UK

Jilian KOHLER, Director, WHO Collaborating Centre for Governance, Transparency & Accountability in the Pharmaceutical Sector, Leslie Dan Faculty of Pharmacy, University of Toronto, Ontario, Canada

Tim MACKEY, Associate Professor, UC San Diego, School of Medicine, Director - Healthcare Research & Policy, UC San Diego – Extension, Director, Global Health Policy Institute, USA

Sarah Harris STEINGRUBER, Programme Manager, Pharmaceuticals & Healthcare Programme, Transparency International, London, UK

Monica Twesiime KIRYA, Senior Advisor, U4 Anti-Corruption Resource Centre, Chr. Michelsen Institute, Bergen, Norway

Taryn VIAN, Clinical Professor of Global Health, Boston University School of Public Health, Boston, MA USA

Paul VINCKE, Managing Director, European Healthcare Fraud & Corruption Network, Brussels, Belgium Aneta WIERZYNSKA, Senior Advisor, Anticorruption and Impact, Ethics Office, The Global Fund to Fight AIDS, Tuberculosis and Malaria, Geneva, Switzerland

Nathalie DE WULF, External Relations Manager, EHFCN - European Healthcare Fraud and Corruption Network, Brussels, Belgium

**WHO/HQ PARTICIPANTS**

Lara Brearley, Technical Officer, UHC2030 Core Team, Health Governance and Financing Department

David Clarke, Health Systems Adviser, Law and Governance, Health System Governance, Policy and Aid Effectiveness, Health Governance and Financing Department

Ibadat S. Dhillon, Technical Officer, Department for Health Workforce

Adama Diop, Consultant, Gender, Equity and Human Rights Team

Veronica Magar, Team Leader, Gender, Equity and Human Rights Team

Dheepa Rajan, Technical Officer, Health Systems, Health System Governance, Policy and Aid Effectiveness, Health Governance and Financing Department

Rebekah Thomas, Technical Officer, Human Rights, Gender, Equity and Human Rights Team

Gerardo Zamora, Programme Officer, Gender, Equity and Human Rights Team

**References from the Background Paper for a WHO Technical Meeting on Anti-corruption, Transparency, and Accountability**

1. Fryatt R, Bennett S, Soucat A. Health sector governance: should we be investing more? BMJ Glob Health. 2017;2(2):e000343.

2. Inter-Agency and Expert Group on Sustainable Development Goal Indicators. Report of the Inter-Agency and Expert Group on Sustainable Development Goal Indicators. E/CN.3/2016/2 [http://unstats.un.org/unsd/statcom/47th-session/documents/2016-2-IAEG-SDGs-E.pdf]. 2015.

3. United Nations Human Rights Council. Final report of the Human Rights Council Advisory Committee on the issue of the negative impact of corruption on the enjoyment of human rights. In: Assembly UNG, ed. A/HRC/28/73. Geneva, Switzerland: UN; 2015:16.

4. United Nations Office of the High Commissioner for Human Rights. Human Rights and anti-corruption. 2018; http://www.ohchr.org/EN/Issues/Development/GoodGovernance/Pages/AntiCorruption.aspx. Accessed 12 March 2008.

5. WHO. Report on the WHO technical meeting on advancing a WHO approach to support member states to strengthen transparency and accountability in health systems. Geneva, Switzerland: WHO;2017.

6. Couffinhal A, Frankowski A. Wasting with intention: Fraud, abuse, corruption and other integrity violations in the health sector. In: OECD, ed. Tackling Wasteful Spending on Health. Paris: OECD Publishing; 2017:265–301.

7. OECD. Consequences of Corruption at the Sector Level and Implications for Economic Growth and Development. Paris: OECD Publishing; 2015.

8. Ecorys. Updated Study of Corruption in the Health Sector. Final Report. Brussels: European Commission;2017.

9. Ecorys. Study on Corruption in the Healthcare Sector. HOME/2011/ISEC/PR/047-A2. October 2013. Luxembourg: Publications Office of the European Union;2013.

10. Hussmann K. Vulnerabilities to corruption in the health sector: Perspectives from Latin American sub-systems for the poor (with a special focus on the sub-national level). Panama: UNDP Regional Centre for Latin America and the Caribbean;2011.

11. Savedoff WD. Transparency and corruption in the health sector: a conceptual framework and ideas for action in Latin America and the Caribbean. Washington, DC: Inter-American Development Bank;2007.

12. Hussmann K. Addressing corruption in the health sector: securing equitable access to health care for everyone. Bergen, Norway: Chr. Michelsen Institute, U4 Anti-corruption Resource Centre;2011.

13. Kohler JC. Fighting corruption in the health sector: methods tools and good practices. New York: UNDP;2011.

14. Mackey TK, Kohler JC, Savedoff WD, et al. The disease of corruption: views on how to fight corruption to advance 21st century global health goals. BMC Med. 2016;14(1):149.

15. Miller W. Corruption and corruptibility. World Development. 2006;34(2):371–380.

16. Habibov N. Effect of corruption on healthcare satisfaction in post-soviet nations: a cross-country instrumental variable analysis of twelve countries. Social Science & Medicine. 2016;152:119–124.

17. Habibov N, Cheung A. Revisiting informal payments in 29 transitional countries: The scale and socio-economic correlates. Social Science & Medicine. 2017;178:28–37.

18.Khodamoradi A, Ghaffari M, Daryabeygi-Khotbehsara R, Sajadi H, Majdzadeh R. A systematic review of empirical studies on methodology and burden of informal patient payments in health systems. International Journal of Health Planning and Management. 2017.

19.Gaffey C. Why the U.S. suspended $21 million in health aid to Kenya. Newsweek. 10 May 2017, 2017.

20. Kamara P. Audit report unearth corruption at Health Ministry. Concord Times. 6 February 2018, 2018.

21.Guinea implements a biometric identification system to conduct a census of civil servants [press release]. www.worldbank.org: World Bank, 3 February 2015 2015.

22.Belita A, Mbindyo P, English M. Absenteeism amongst health workers: Developing a typology to support empiric work in low-income countries and characterizing reported associations. Human Resources for Health. 2013;11:34.

23.Audit Service of Sierra Leone. Report on the audit of management of the Ebola funds, May-Oct. 2014. 2014.

24.Keta E. Patients pay price for Albania’s drug reform. BalkanInsight. 24 September 2015, 2015.

25.Waning B, Vian T. Transparency and accountability in an electronic era: the case of pharmaceutical procurements. U4 Brief 10. Bergen, Norway: U4 Anti-corruption Resource Centre;2008.

26.Wojczewski S, Willcox M, Mubangizi V, et al. Portrayal of the human resource crisis and accountability in healthcare: a qualitative analysis of ugandan newspapers. PloS one. 2015;10(4):e0121766.

27.Kagotho N, Bunger A, Wagner K. ‘They make money off of us’: a phenomenological analysis of consumer perceptions of corruption in Kenya’s HIV response system. BMC Health Serv Res. 2016;16:468.

28.Vian T, Gryboski K, Sinoimeri Z, Hall R. Informal payments in government health facilities in Albania: results of a qualitative study. Soc Sci Med. 2006;62(4):877–887.

29.Transparency International. Global Corruption Barometer: Citizens voices from around the world. 2017; https://www.transparency.org/news/feature/global_corruption_barometer_citizens_voices_from_around_the_world. Accessed 4 January 2018.

30.TNS Opinion & Social. Special Eurobarometer 470 Corruption Report. TNS Opinion & Social, at the request of the European Commission, Directorate-General for Home Affairs;2017.

31.Andersson N. Building the community voice into planning: 25 years of methods development in social audit. BMC Health Serv Res. 2011;11 Suppl 2:S1.

32.Arostegui J, Hernandez C, Suazo H, et al. Auditing Nicaragua’s anti-corruption struggle, 1998 to 2009. BMC Health Serv Res. 2011;11 Suppl 2:S3.

33.Mejsner SB, Karlsson LE. Informal patient payments and bought and brought goods in the Western Balkans–a scoping review. International Journal of Health Policy and Management. 2017;6(11):621–637.

34.Kankeu HT, Ventelou B. Socioeconomic inequalities in informal payments for health care: An assessment of the ‘Robin Hood’ hypothesis in 33 African countries. Soc Sci Med. 2016;151:173–186.

35.Serneels P, Lievens T. Microeconomic institutions and personnel economics for health care delivery: a formal exploration of what matters to health workers in Rwanda. Hum Resour Health. 2018;16(1):7.

36.Rashidian A, Joudaki H, Vian T. No evidence of the effect of the interventions to combat health care fraud and abuse: a systematic review of literature. PloS one. 2012;7(8):e41988.

37.Berwick DM, Hackbarth AD. Eliminating waste in US health care. JAMA. 2012;307(14):1513–1516.

38.Gee J, Burton B. The financial cost of healthcare fraud 2015: What data from around the world shows. PKF Littlejohn LLP;2015.

39.Jesilow P, Burton B. Detecting healthcare fraud and abuse in the USA. Oxford Research Encyclopedia of Criminology. 2017.

40.Russo G, McPake B, Fronteira I, Ferrinho P. Negotiating markets for health: an exploration of physicians’ engagement in dual practice in three African capital cities. Health Policy Plan. 2014;29(6):774–783.

41.Berman P, Cuizon D. Multiple public-private jobholding of health care providers in developing countries: an exploration of theory and evidence. London: Department for International Development Health Systems Resource Center;2004.

42.Abera GG, Alemayehu YK, Herrin J. Public-on-private dual practice among physicians in public hospitals of Tigray National Regional State, North Ethiopia: perspectives of physicians, patients and managers. BMC Health Serv Res. 2017;17(1):713.

43.Ashmore J, Gilson L. Conceptualizing the impacts of dual practice on the retention of public sector specialists - evidence from South Africa. Hum Resour Health. 2015;13:3.

44.Johnston A, Holt DW. Substandard drugs: a potential crisis for public health. British Journal of Clinical Pharmacology. 2014;78(2):218–243.

45.Hamilton WL, Doyle C, Halliwell-Ewen M, Lambert G. Public health interventions to protect against falsified medicines: a systematic review of international, national and local policies. Health Policy and Planning. 2016;31:1448–1466.

46.Renschler JP, Walters KM, Newton PN, Laxminarayan R. Estimated under-five deaths associated with poor-quality antimalarials in sub-Saharan Africa. Am J Trop Med Hyg. 2015;92(6 Suppl):119–126.

47.Almuzaini T, Choonara I, Sammons H. Substandard and counterfeit medicines: a systematic review of the literature. BMJ Open. 2013;3(8):e002923.

48.Bate R, Mathur A. Corruption and medicine quality in Latin America: a pilot study. Pharmacy (Basel). 2018.

49.Sahoo J, Mahajan PB, Paul S, Bhatia V, Patra AK, Hembram DK. Operational Assessment of ICDS Scheme at Grass Root Level in a Rural Area of Eastern India: Time to Introspect. J Clin Diagn Res. 2016;10(12):LC28-LC32.

50.Davis J. Corruption in Public Service Delivery: Experience from South Asia’s Water and Sanitation Sector. World Development. 2004;32(1):53–71.

51.Uchendu FN, Abolarin TO. Corrupt practices negatively influenced food security and live expectancy in developing countries. Pan Afr Med J. 2015;20:110.

52.Green P. Disaster by design: corruption, construction, and catastrophe. The British Journal of Criminology. 2005;45(4):528–546.

53.Bloom D, Fink G. The economic case for devoting public resources to health. In: Farrar J, Hotez P, Junghanss T, Kang G, Laloo D, White N, eds. Manson’s Tropical Diseases. 23rd ed. Philadelphia, PA: Sanders; 2014.

54.Jamison DT, Summers LH, Alleyne G, et al. Global health 2035: A world converging within a generation. The Lancet. 2013;382(9908):1898–1955.

55.Hanf M, Van-Melle A, Fraisse F, Roger A, Carme B, Nacher M. Corruption kills: Estimating the global impact of corruption on children deaths. PloS one. 2011;6(11):e26990.

56.Muldoon KA, Galway LP, Nakajima M, et al. Health system determinants of infant, child and maternal mortality: A cross-sectional study of UN member countries. Globalization and health. 2011;7:42.

57.Ciccone DK, Vian T, Maurer L, Bradley EH. Linking governance mechanisms to health outcomes: A review of the literature in low- and middle-income countries. Social science & medicine. 2014;117:86–95.

58.Fagan C. The anti-corruption catalyst: Realising the MDGs by 2015. Berlin: Transparency International;2010.

59.Witvliet MI, Kunst AE, Arah OA, Stronks K. Sick regimes and sick people: a multilevel investigation of the population health consequences of perceived national corruption. Tropical medicine & international health: TM & IH. 2013;18(10):1240–1247.

60.Baji P, Pavlova M, Gulácsi L, Groot W. Changes in equity in out-of-pocket payments during the period of health care reforms: evidence from Hungary. International Journal for Equity in Health. 2012;11:36.

61.Kaitelidou D, Tsirona CS, Galanis PA, et al. Informal payments for maternity health services in public hospitals in Greece. Health Policy. 2013;109(1):23–30.

62.Vian T, Feeley FG, Domente S, Negruta A, Matei A, Habicht J. Barriers to universal health coverage in Republic of Moldova: a policy analysis of formal and informal out-of-pocket payments. BMC Health Serv Res. 2015;15:319.

63.Usher AD. Donors lose faith in Zambian Health Ministry. The Lancet. 2010;376:403–404.

64.Savedoff W, Glassman A, Madan J. Global Health, Aid and Corruption: Can We Escape the Scandal Cycle? CGD Policy Paper 086. June 2016. Washington, DC: Center for Global Development;2016.

65.Transparency International. What is corruption? 2017; https://www.transparency.org/what-is-corruption. Accessed 4 January 2017.

66.United Nations Convention Against Corruption. Convention against corruption. https://www.unodc.org/unodc/en/treaties/CAC/Accessed 4 January 2018.

67.Dionne KY. Doomed Interventions: the Failure of Global Responses to AIDS in Africa. UK: Cambridge University Press;2018.

68.Savedoff W, Hussmann K. Why are health systems prone to corruption? In: Transparency International, ed. Global Corruption Report 2006: Special Focus on Corruption and Health. London, UK: Pluto Press; 2006:4–16.

69.Piotrowski SJ. Transparency: a regime value linked with ethics. Administration & Society. 2014;46(2):181–189.

70.Vian T. Exploring the construction of transparency: an analysis of health managers’ narratives. Global Health Governance. 2012;V(2):1–24.

71.Paschke A, Dimancesco D, Vian T, Kohler JC, Forte G. A practical approach to strengthening transparency and accountability in national pharmaceutical systems.

72.Lambert-Mogiliansky A. Social accountability to contain corruption. Journal of Development Economics. 2015;116:158–168.

73.Piotrowski SJ, Borry E. An analytical framework for open meetings and transparency. Public Administration and Management. 2010;15(1):138–176.

74.Vian T, Kohler JC, Forte G, Dimancesco D. Promoting transparency, accountability, and access through a multi-stakeholder initiative: lessons from the medicines transparency alliance. J Pharm Policy Pract. 2017;10:18.

75.Transparency International. Global Corruption Report 2006: Special Focus Corruption and Health. London: Pluto Press; 2006.

76.Vian T. Review of corruption in the health sector: theory, methods and interventions. Health Policy Plan. 2008;23(2):83–94.

77.Hernández-Aguado L, E C-R. Pathways of undue influence in health policy-making: a main actor’s perspective. Journal of Epidemiology and Community Health. 2018;72(2):154–159.

78.Graycar A, Prenzler T. Understanding and preventing corruption. London: Palgrave Macmillan; 2013.

79.Vian T, Savedoff W, Mathisen H. Anticorruption in the Health Sector: Strategies for Transparency and Accountability. Herndon, VA: Kumarian Press; 2010.

80.Brinkerhoff D. Accountability and health systems: toward conceptual clarity and policy relevance. Health Policy & Planning. 2004;19(6):371–379.

81.Brinkerhoff D, Bossert T. Health governance: principal-agent linkages and health system strengthening. Health Policy & Planning. 2014;29(6):685–693.

82.Buckland-Merrett G, Kilkenny C, Reed T. Civil society engagement in multi-stakeholder dialogue: a qualitative study exploring the opinions and perceptions of MeTA members. J Pharm Policy Pract. 2017;10:5.

83.Baez Camargo C. Using power and influence analysis to address corruption risks: the case of Ugandan drug supply. U4 Brief 2012:6. Chr Michelsen Institute, U4 Anti-corruption Resource Centre2012.

84.Peixoto T, J F. When does ICT-enabled citizen voice lead to government responsiveness? IDS Bulletin. 2016;47(1).

85.Molina E, Carella L, Pacheco A, Cruces G, Gasparini L. Community monitoring interventions to curb corruption and increase access and quality of service delivery in low- and middle-income countries. Oslo, Norway: The Campbell Collaboration;2016.

86.Heald D. Varieties of transparency. In: Heald D, Hood C, eds. Transparency: The Key to Better Governance? New York: Oxford University Press; 2006:25–43.

87.Oliver R. What is Transparency? New York: McGraw-Hill; 2004.

88.Vian T. Exploring the construction of transparency: an analysis of health managers’ narrative. Global Health Governance. 2012;V(2):1–24.

89.Bruckner T. Clinical trial transparency: a guide for policy makers. UK: Transparency International Pharmaceuticals & Healthcare Programme;2017.

90.Buvinic M, O’Donnell M. Revisiting what works: women, economic empowerment and smart design. Washington, DC: Center for Global Development;2016.

91.Brinkerhoff D, Jacobstein D, Kanthor J, Rajan D, Shepard K. Accountability, health governance, and health systems: uncovering the linkages. Washington, DC: Health Finance & Governance Project for USAID;2017.

92.Baldridge A, Elfman E, Heredia-Ortiz E, Barroy H, Saleh K, Connor C. Public financial management, health governance, and health systems. Washington, DC: Health Finance & Governance Project for USAID;2017.

93.Gaitonde R, Oxman A, Okebukola P, G R. Interventions to reduce corruption in the health sector (Review). Cochrane Database of Systematic Reviews. 2016(8):CD008856.

94.Vian T. Corruption risk assessment in the health sector in Kosovo. Pristina, Kosovo: UNDP;2014.

95.Management Systems International. Rwanda Health Sector Corruption Risk Assessment. Washington, DC: MSI for USAID;2008.

96.Fight against corruption and Fostering Good Governance/Fight against money-laundering (PGG-Regional) [press release]. Strasbourg, France: Council of Europe2017.

97.Vian T. Implementing a transparency and accountability policy to reduce corruption: The GAVI Alliance in Cameroon. U4 Brief 2013:9). Bergen, Norway: Chr. Michelsen Institute, U4 Anti-Corruption Resource Centre;2013.

98.The Global Fund. The Global Fund Policy to Combat Fraud and Corruption. GF/B38/06, Annex 4. Approved by the Board during the 38th Board Meeting on 15 November 2017. Geneva, Switzerland: The Global Fund;2017.

99.Feeley F. Implications for corruption control of laws and regulations governing health insurance in Albania. Technical Paper. Project Against Corruption in Albania (PACA). Strasbourg, France: Council of Europe;2012.

100.Medicines Transparency Alliance. Data Disclosure Survey. 2018; http://www.medicinestransparency.org/?id=588. Accessed 23 February 2018.

101.Martin J, Ollier L. Evaluation of the Good Governance for Medicines Programme (2004 – 2012): Brief summary of findings. WHO/EMP/MPC/2013.1. Geneva, Switzerland: World Health Organization;2013.

102.Ash Center for Democratic Governance and Innovation. Citizen voices, community solutions: designing better transparency and accountability approaches to improve health. Cambridge, MA: Harvard Kennedy School;2017.

103.IIEP-UNESCO. 10 ways to promote transparency and accountability in education. 2018; http://www.iiep.unesco.org/en/10-ways-promote-transparency-and-accountability-education-4307. Accessed 28 January 2018.

104.International Budget Partnership. It’s Our Money Where’s It Gone. 2009.

105.Social Justice Coalition, Ndifuna Ukwazi, International Budget Partnership. A guide to conducting social audits in South Africa. Washington, DC: International Budget Partnership;2015.

106.Asha G, Sri BS, Rajani V. Strengthening people-centered services through improved accountability: the Enabling Community Action for Maternal Health Project in Gujarat, India. Gujarat, India: SAHAJ;2016.

107.Vian T. Complaint mechanisms in health organizations. U4 brief 2013:6. Bergen, Norway: Chr. Michelsen Institute, U4 Anti-corruption Resource Centre;2013.

108.Huss R, Green A, Sudarshan H, et al. Good governance and corruption in the health sector: lessons from the Karnataka experience. Health Policy Plan. 2011;26(6):471–484.

109.Transparency in Health Engagement Project. Baseline assessment of High State Audit, Ombudsperson, HIDACCI, civil society and media organizations in Albania. Tirana, Albania: University Research Corporation with Boston University and Tetra Tech, for USAID;2017.

110.Thomas AR. Justice at last for thousands killed by Ebola negligence and corruption in Sierra Leone. The Sierra Leone Telegraph. 15 December 2017, 2017.

111.Asiedu KF, Deffor EW. Fighting corruption by means of effective internal audit function: Evidence from the Ghanaian public sector. International Journal of Auditing. 2017;21:82–99.

112.Religioni U, Swieczkowski D, Gawronska A, et al. Hospital Audit as a Useful Tool in the Process of Introducing Falsified Medicines Directive (FMD) into Hospital Pharmacy Settings-A Pilot Study. Pharmacy (Basel). 2017;5(4).

113.Chereches RM, Ungureanu MI, Sandu P, Rus IA. Defining informal payments in healthcare: a systematic review. Health Policy. 2013;110(2–3):105–114.

114.Stepurko T, Pavlova M, Gryga I, Groot W. Empirical studies on informal patient payments for health care services: a systematic and critical review of research methods and instruments. BMC Health Serv Res. 2010;10:273.

115.Weiss B, Pollack AA. Barriers to global health development: an international quantitative survey. PloS one. 2017;12(10):e0184846.

116.Slack D. VA bosses in 7 states falsified vets’ wait times for care. USA Today. 7 April 2016.

117.World Health Organization. Framework on integrated, people-centred health services. Report by the Secretariat. World Health Assembly document A69/39. Geneva, Switzerland: WHO;2016.

118.USAID. Health System Assessment Approach: A How-to Manual Version 3.0. Washington, DC: USAID;2017.

119.World Health Organization. Global Atlas of Medical Devices. Geneva, Switzerland: WHO; 2017.

120.Schmets G, Rajan D, Kadandale S, eds. Strategizing National Health in the 21st Century: a Handbook. Geneva: World Health Organization; 2016.
